# First detection and phylogenetic analysis of porcine circovirus type 2 in raccoon dogs

**DOI:** 10.1186/s12917-019-1856-2

**Published:** 2019-04-08

**Authors:** Tao Song, Jianxiang Hao, Ran Zhang, Menghu Tang, Wenao Li, Weirong Hui, Qiyuan Fu, Chunfang Wang, Shuyang Xin, Shoucong Zhang, Ping Rui, Hai Ren, Zengjun Ma

**Affiliations:** 1 Hebei Key Laboratory of Preventive Veterinary Medicine, College of Animal Science and Technology, Hebei Normal University of Science and Technology, Qinhuangdao, 066004 China; 2 College of Life Sciences, Hebei Normal University, Shijiazhuang, 050024 China

**Keywords:** Raccoon dogs, Porcine circovirus type 2, Phylogenetic analysis

## Abstract

**Background:**

Porcine circovirus type 2 (PCV2) is a major emerging virus of porcine circovirus-associated disease (PCVAD), which has brought huge economic losses to the global pig industry. Pigs are well known as the natural reservoir of PCV2. Recently, many researchers have revealed PCV2 could infect many other mammals like mice, calves, minks, dogs and goats. In 2018, our laboratory has admitted six cases of raccoon dogs from Qinhuangdao city of China, which were characterized by inappetence, lethargy, depression, abortion, and sterility.

**Results:**

At last, six raccoon dog-origin PCV2 strains were isolated in this study. Pairwise-sequence comparisons demonstrated that the six raccoon dog-origin PCV2 strains shared a nucleotide similarity of 92.1–99.8% among 40 PCV2 representative strains. Phylogenetic analysis indicated these PCV2 isolates belonged to Chinese epidemic genotypes PCV2b and PCV2d. And aborted or sterile symptom was significantly associated with PCV2 infection in raccoon dogs by the chi-square test (χ^2^ = 87.3, *p* < 0.001). The retrospective study revealed that raccoon dog-origin PCV2 strains shared 100% sequence similarity with the PCV2 stains isolated from pig farms around these raccoon dog farms, respectively.

**Conclusion:**

In this study, the first supported evidence of PCV2 prevalence in raccoon dog farms of China was documented. PCV2 may be one of the most significant causative agents resulting in the reproductive failure of farmed raccoon dogs, implying that PCV2 could transmit from pigs to raccoon dogs. That indicated that PCV2 cross-species transmission will be a serious threat to China’s fur animal farming industry.

## Background

After 2010, China has increased to become one of the biggest fur animal farming countries. But poor feeding condition is still the serious adverse factor of restricting China’s fur animal farming industry [1]. Furthermore, the small-scale family breeding model is still the dominant breeding model of fur animals in China. Many cross-species diseases from other breeding animals could easily attack fur animals, especially those from surrounding pig farms [2–4]. Porcine circoviruses (PCV) belong to the *Circovirus* genus within the *Circoviridae* family, which are small non-enveloped single-strand circular DNA viruses. They have been firstly identified in 1982, and contain three types, including PCV1, PCV2 and PCV3 [5, 6].

PCV1 is known to have no association with clinical diseases [7]. PCV3 has recently been first identified in the USA in pigs with characteristics of cardiac and multisystemic inflammation with metagenomic sequencing [8, 9]. PCV2 has been recognized as one of the main agents responsible for PCV-associated disease (PCVAD) which has caused huge economic losses to the global pig industry [10, 11]. PCV2 has been currently classified into six major genotypes (PCV2a, PCV2b, PCV2c, PCV2d, PCV2e and PCV2f) [12–14], of which PCV2b and PCV2d genotypes are the predominant strains in China [15–17]. The complete genome of PCV2 ranges from 1766 or 1768 bp and encodes at least 11 predicted open reading frames (ORFs) [18, 19]. To date, PCV2 was reported to be discovered in many other reservoirs such as mice, calves, minks, dogs, foxes and goats, besides pigs as its natural reservoirs [4, 20–26]. In this study six PCV2 isolates were identified from farmed raccoon dogs with the symptom of reproductive failure. These findings suggested the possibility of PCV2 cross-species transmission. And this is the first time to report the genetic analysis of raccoon dog-origin PCV2 isolates.

## Methods

### Samples treatment

In 2018, our laboratory has admitted six cases of raccoon dogs from Qinhuangdao, China with clinical symptoms of inappetence, lethargy, depression, abortion, and sterility. After collecting raccoon dog samples, bacterial culture was immediately carried out. The lymph nodes and spleens of all these cases were cultured onto both blood agar plates and tryptic soy agar plates at 37 °C for 24–48 h under the aerobic and anaerobic conditions respectively. In addition, all the above tissues were aseptically collected and homogenized by TissueLyser II (QIAGEN, Germany), then centrifuged at 8000 *g* for 10 min for further study.

### Viral genome extract and detection

Special detecting primers were synthesized to detect the common viruses infected raccoon dogs such as canine parvovirus (CPV), canine adenovirus (CAV), canine distemper virus (CDV), Pseudorabies virus (PRV), Hepatitis E Virus (HEV) and PCV2, as described previously [15, 27–31]. Viral DNA or RNA was extracted from each samples using a TIANamp virus DNA/RNA extraction kit (Tiangen, Beijing) following the manufacturer’s instructions.

RT-PCR was carried out to detect CDV and HEV with the PrimeScript One Step RT-PCR kit (TaKaRa, Dalian) following the manufacturer’s instructions. RT-PCR conditions comprised 50 °C for 30 min, 94 °C for 2 min, 30 cycles of denaturation at 94 °C for 30 s, annealing at 56 °C for 30 s, and extension at 72 °C for 30 s, ended with a final extension at 72 °C for 10 min. PCR was carried out to detect CAV, CPV, PRV, and PCV2 with *Ex Taq* DNA Polymerase (TaKaRa, Dalian) following the manufacturer’s instructions. The PCR conditions comprised 95 °C for 5 min, 30 cycles of denaturation at 95 °C for 30 s, annealing at 55 °C for 30 s, and extension at 72 °C for 30 s, ended with a final step at 72 °C for 10 min. RT-PCR and PCR products were run on a 1% agarose gel and imaged under ultraviolet light.

### Viral isolation and indirect immunofluorescence assay (IFA)

Porcine Kidney Epithelial (PK-15) cells were purchased from American Type Culture Collection (ATCC CCL-33), and cells between passages 21 to 25 were used for experiments. The tissue samples of raccoon dogs were treated and cultured on PK-15 cells for three serial passages. Then the cell lysates were infected on PK-15 cells for IFA assay to detect PCV2, as described previously [26]. Finally, the IFA results were observed using inverted fluorescent microscope (Olympus IX73). The PCV2 strain Hebei2 (GenBank no. MG1825) preserved in our laboratory was infected with PK-15 cells as a positive control, while mock-infected PK-15 cells as a negative control.

### Viral genome sequencing

The PCV2 genome sequences were amplified using a pair of special primers, as described previously [32]. PCR was performed using the following conditions: at 95 °C for 5 min, followed by 33 cycles of denaturation at 95 °C for 1 min, annealing at 54 °C for 1 min, and extension at 72 °C for 2 min, and ended with a final step at 72 °C for 10 min. PCR products of PCV2 fragments were purified with Gel Extraction Kit (Tiangen, Beijing) and subcloned into the pMD18-T vector (TaKaRa, Dalian). The recombinant plasmids were identified by enzyme digestion and sequenced using Sanger sequencing (Augct DNA-Syn Biotechnology, Beijing).

### Multiple sequences comparison and phylogenetic analyses

The complete sequences of all PCV2 stains were assembled using the SeqMan v7.1.0 program (Lasergene, DNAStar, USA), and sequence homology analysis was performed using the MegAlign v7.1.0 program (Lasergene, DNAStar, USA). Phylogenetic tree was constructed using the neighbour-joining method of MEGA 7.0 software [33]. The bootstrap consensus tree inferred from 1000 replicates is taken to represent the evolutionary history, and the neighbour-joining method was used to infer the evolutionary history [34, 35].

### The retrospective and tracing investigations

Three hundred serum samples of raccoon dogs were collected from eight raccoon dog farms in Qinhuangdao, which included 150 samples with no symptoms and 150 samples with symptoms of abortion or sterility. A real-time SYBR green PCR was performed to quantify PCV2 in these serum samples, as described previously [36]. Cycling conditions were 95 °C for 10 min, followed by 40 cycles of denaturation at 95 °C for 30 s, annealing at 59 °C for 10 s, and extension at 72 °C for 30 s. Fluorescence normalization and data analysis were performed using the ABI Prism 7500 (Applied Biosystems Inc., USA). A chi-square test was used to evaluate the association between aborted or sterile symptom and PCV2 infection in raccoon dogs.

For tracing the origin of the PCV2 stains isolated from raccoon dogs, 210 pig serum samples from 21 pig farms (10 samples per farm) around the six raccoon dog farms were collected and detected for PCV2 by PCR assay. One positive sample from every PCV2-positive farm was selected to amplify the PCV2 complete genome as above. PCR fragments of PCV2 were purified, cloned, sequenced, and assembled as above. Multiple sequence alignments were generated using the MegAlign v7.1.0 program. Sequence similarity analysis demonstrated the sequence distances between pig-origin PCV2 strains and raccoon dog-origin PCV2 strains.

## Results

### Sample detecting

Bacterial culture revealed no common bacterium was observed on the plates cultivated with tissue samples of these raccoon dogs. The (RT)-PCR results proven all samples negative for CDV, CAV, CPV, HEV, and PRV using specific detecting primers to these viruses, but all samples positive for PCV2.

### Virus isolation and identification

The tissue homogenates of raccoon dogs were subsequently inoculated on PK-15 cells. After three blind passages, no cytopathic effect (CPE) was observed. However, PCR detection showed all these passage cultures were PCV2-positive. Further IFA experiment demonstrated all PK-15 cells infected with these viral cultures could cross-react with anti-PCV2 Mab, implying that PCV2 strains were isolated from these raccoon dogs. At last, the complete sequences of raccoon dog-origin PCV2 isolates were designated as Rac-hb1~Rac-hb6 respectively.

### Genome sequencing of raccoon dog-origin PCV2 strains

The fragments of PCV2 complete genome sequences were amplified as described previously [17]. After sequence assembling, the complete genome sequences of strains Rac-hb1~Rac-hb6 were obtained with all 1767 nt in length, and deposited into the GenBank database under accession numbers MH373555~MH373560 respectively.

### Multiple sequences comparison and phylogenetic analyses

**Fig. 1:**
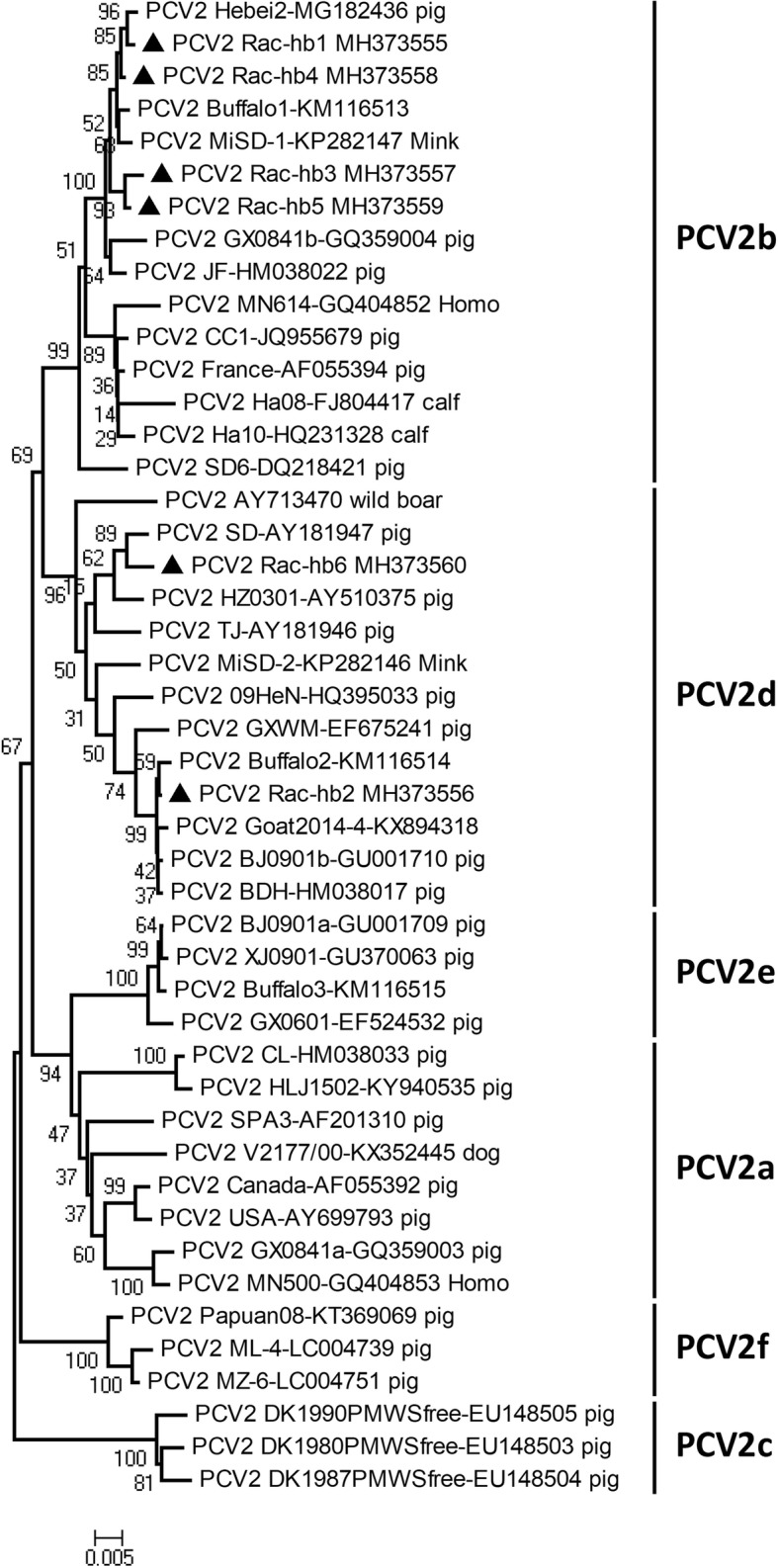
Phylogenetic tree of the complete genome sequences of PCV2. The tree was constructed using MEGA 7.0 software and analyzed by neighbour-joining method using 1000 replicates on bootstrap analysis. Six raccoon dog-origin PCV2 strains isolated in this study were labeled with black triangles (▲)

The complete genome sequences of six raccoon dog-origin PCV2 strains showed a high similarity varied from 92.1 to 99.8% compared with those of 40 PCV2 representative strains (Table 1) based on multiple sequences comparison analysis. Considering the phylogenetic analysis of PCV2 complete genome sequences, the six raccoon dog-origin PCV2 strains belong to the epidemic PCV2 strains of China (PCV2b and PCV2d) [15, 17, 37, 38]. Thereinto, strains Rac-hb1, Rac-hb3, Rac-hb4 and Rac-hb5 belong to PCV2b, and strains Rac-hb2 and Rac-hb6 belong to PCV2d (Fig. 1).

**Table 1:** PCV2 representative strains used in this study

Strain name	Accession no.	Source	Genotype	Strain name	Accession no.	Source	Genotype
Canada	AF055392	pig	PCV2a	DK1990PMWSfree	EU148505	pig	PCV2c
GX0841a	GQ359003	pig	PCV2a	MiSD-2	KP282146	mink	PCV2d
MN500	GQ404853	Homo	PCV2a	TJ	AY181946	pig	PCV2d
V2177/00	KX352445	dog	PCV2a	BJ0901b	GU001710	pig	PCV2d
USA	AY699793	pig	PCV2a	German	AY713470	wild boar	PCV2d
SPA3	AF201310	pig	PCV2a	GXWM	EF675241	pig	PCV2d
CL	HM038033	pig	PCV2a	Buffalo2	KM116514	buffalo	PCV2d
HLJ1502	KY940535	pig	PCV2a	BDH	HM038017	pig	PCV2d
France	AF055394	pig	PCV2b	SD	AY181947	pig	PCV2d
Ha08	FJ804417	calf	PCV2b	Goat2014–4	KX894318	goat	PCV2d
GX0841b	GQ359004	pig	PCV2b	HZ0301	AY510375	pig	PCV2d
MN614	GQ404852	Homo	PCV2b	09HeN	HQ395033	pig	PCV2d
SD6	DQ218421	pig	PCV2b	GX0601	EF524532	pig	PCV2e
Ha10	HQ231328	calf	PCV2b	BJ0901a	GU001709	pig	PCV2e
Buffalo1	KM116513	buffalo	PCV2b	XJ0901	GU370063	pig	PCV2e
JF	HM038022	pig	PCV2b	Buffalo3	KM116515	buffalo	PCV2e
CC1	JQ955679	pig	PCV2b	ML-4	LC004739	pig	PCV2f
MiSD-1	KP282147	mink	PCV2b	MZ-6	LC004751	pig	PCV2f
DK1980PMWSfree	EU148503	pig	PCV2c	Papuan08	KT369069	pig	PCV2f
DK1987PMWSfree	EU148504	pig	PCV2c	Hebei2	MG182436	pig	PCV2b

### The retrospective and tracing investigations of raccoon dog-origin PCV2 strains

**Fig. 2:**
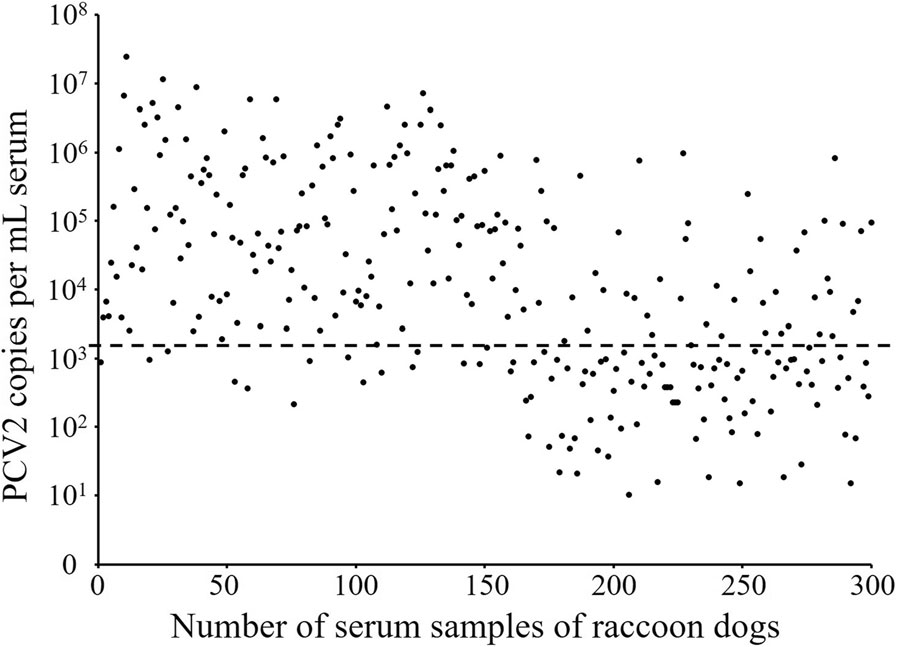
Nucleotide sequence similarity (%) of the complete genome sequences between raccoon dog-origin PCV2 strains and pig-origin PCV2 strains

The retrospective epidemiological study on PCV2-infected raccoon dogs was performed to detect 300 serum samples collected from the raccoon dog farms in Qinhuangdao using real-time PCR. The results demonstrated the positive rate of PCV2 was 63.3% (190/300), including 89.3% (134/150) in diseased raccoon dogs with symptoms of abortion or sterility and 37.3% (56/150) in raccoon dogs with no symptoms (Fig. 2). There was a higher positive rate of PCV2 in the aborted or sterile raccoon dogs, while a lower positive rate of PCV2 in healthy raccoon dogs. The Chi-square test of significant difference (χ^2^ = 87.3, *p* < 0.001) revealed that aborted or sterile symptom was significantly associated with PCV2 infection in raccoon dogs.

**Fig. 3:**
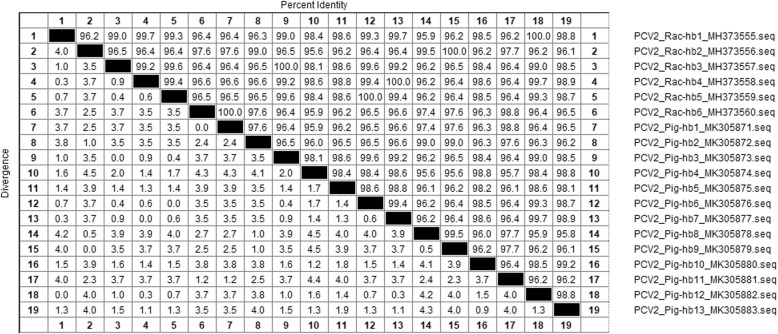
PCV2 copies per mL serum of raccoon dogs followed by real-time PCR on the quantification of PCV2 viral DNA. The lower detection limit (2.2 × 10^3^ copies) is generated based on the standard curve (the broken line)

The tracing study revealed that 13 genome sequences of pig-origin PCV2 isolates were obtained and designated as Pig-hb1~Pig-hb13 (GenBank accession no. MK305871~MK305883). Sequence similarity analysis showed that the complete genomes of six pig-origin PCV2 strains (Pig-hb12, Pig-hb9, Pig-hb3, Pig-hb7, Pig-hb6, and Pig-hb1) were shared 100% nucleotide identity with those of strains Rac-hb1~Rac-hb6, respectively (Fig. 3).

## Discussion

PCV2 is the key agents responsible for PCVAD in pigs, including several clinical syndromes such as postweaning multisystemic wasting syndrome (PMWS), porcine dermatitis and nephropathy syndrome (PDNS), porcine respiratory disease complex (PRDC) and PCV2-associated reproductive failure [39, 40]. Recent studies have suggested that PCV2 was detected in non-porcine species with no symptoms or mild diarrhea [4, 21, 24, 25, 41]. Surprisingly, we firstly detected and isolated PCV2 from raccoon dogs with reproductive failure. Phylogenic analysis indicated that these raccoon dog-origin PCV2 isolates were confirmed to belong to the genotypes PCV2b and PCV2d, which were the dominating PCV2 genotypes in current China [15, 17, 37, 38].

The retrospective investigation has revealed that aborted or sterile symptom was significantly associated with PCV2 infection in raccoon dogs. This indicated that PCV2 may be one of the most significant causative agents resulting in the reproductive failure of farmed raccoon dogs. However, further animal experiment needs to be validate the effect on PCV2 infection in raccoon dogs during oestrus.

The tracing study has demonstrated these raccoon dog-origin PCV2 strains originates from pig farms around raccoon dog farms. Previous studies have also revealed that PCV2 could be detected and isolated from minks and foxes in China, implying that PCV2 becomes an increasing threat to China’s fur animal farming industry [4, 26]. And horizontal transmission especially contact between animals proved to be the main route of PCV2 transmission [42]. What’s more, the small-scale family breeding model of fur animals in China, mixed-breeded with other different livestock, has increased the risk of PCV2 cross-species transmission. Therefore, in order to prevent PCV2 cross-species transmission, very strong biosecurity barriers should be built between fur-animal farms and pig farms.

## Conclusion

In summary, this study has provide first evidence of PCV2 prevalence in raccoon dogs in China. The genetic analysis and epidemiological investigation of raccoon dog-origin PCV2 stains in this research will help enrich the data of PCV2 cross-species transmission. Much attention should be paid to the cross-species infectious mechanism of PCV2 and the commercial PCV2 vaccine for raccoon dogs.
